# Prevalence of metabolic components in university students^1^

**DOI:** 10.1590/0104-1169.0129.2514

**Published:** 2014

**Authors:** Ana Roberta Vilarouca da Silva, Luana Savana Nascimento de Sousa, Telma de Sousa Rocha, Ramiro Marx Alves Cortez, Layla Gonçalves do Nascimento Macêdo, Paulo César de Almeida

**Affiliations:** 2 PhD, Professor, Departamento de Enfermagem, Universidade Federal do Piauí, Picos, PI, Brazil; 3 RN; 4 RN, Prefeitura Municipal de São Raimundo Nonato, São Raimundo Nonato, PI, Brazil; 5 RN, Prefeitura Municipal de Santa Cruz, Santa Cruz, PI, Brazil; 6 RN, Prefeitura Municipal de Itainópolis, Itainópolis, PI, Brazil; 7 PhD, Professor, Departamento de Enfermagem, Universidade Estadual do Ceará, Fortaleza, CE, Brazil

**Keywords:** Students, Metabolic Syndrome x, Risk Factors

## Abstract

**OBJECTIVE:**

to identify the frequency of components of Metabolic Syndrome (MetS) among university students.

**METHOD:**

descriptive study with 550 students, from various courses run by a public university. The socioeconomic data, lifestyle, and components of MetS were filled out using a questionnaire. Blood sample collection was undertaken in the university itself by a contracted clinical analysis laboratory.

**RESULTS:**

66.2% were female, with a mean age of 22.6±4.41; 71.7% were sedentary; 1.8% stated that they smoke; and 48.5% were classified as at medium risk for alcoholism. 5.8% had raised abdominal circumference and 20.4% had excess weight; 1.3% and 18.9% had raised fasting blood glucose levels and triglycerides, respectively; 64.5% had low HDL cholesterol and 8.7% had blood pressure levels compatible with borderline high blood pressure. Thus, of the sample, 64.4% had at least one component for MetS; 11.6% had two, and 3.5% had three or more.

**CONCLUSION:**

a significant proportion of the population already has the components for metabolic syndrome, and this profile reinforces the importance of early diagnosis so as to reduce the risk of developing chronic comorbidities.

## Introduction

In Brazil, the frequency of Metabolic Syndrome (MetS) is unknown in various regions, and little studied in differing populations. This is because it arises from globalization, an inherent indicator of modification to society's lifestyle. 

Its development in an individual depends on the complex interaction between genetic predisposition and factors linked to lifestyle, such as dietary standards, sedentarism and obesity^(^ 1 ^)^.

The physical pathology of MetS has various origins, but sedentarism and obesity, associated with dietary standards and heredity factors, are strong interactions for its development. One of the most frequently-used definitions of MetS is that proposed by the Expert Panel on Detection, Evaluation, and Treatment of High Blood Cholesterol in Adults^(2) ^and is based on the presence of three or more cardiovascular risk factors in an individual: abdominal obesity, intolerance to glucose, dyslipidemia (a rise in the levels of triglycerides in the plasma and reduction of the levels of High Density Lipoprotein -HDL-c) and increase in systemic arterial hypertension (SAH)^(^ 3 ^)^. 

MetS is responsible for approximately 7% of deaths worldwide, regardless of the cause, and for 17% of those related to cardiovascular diseases (CVD). It increases the risk of CVD by 34% and 16% for men and women, respectively. Taking each component of MetS as a base, the most morbid are raised blood pressure (33%) and low HDL cholesterol (25%)^(^ 4 ^)^.

For young adults, such as, for example, university students, the international literature has evidenced investigations undertaken at the University of Kansas, the University of Carabobo (Venezuela) and the University of Stellenbosch (South Africa), which found significant prevalences of MetS. According to the researchers, it is possible that this is owed to the changes in the lives of individuals on entering university, which can lead to the adoption of inappropriate eating habits which favor the appearance of risk factors for chronic noncommunicable diseases^(^ 5 ^-^ 6 ^)^.

Brazil - and many other countries, worldwide - presented a concerning situation regarding the chronic diseases, not only because of the high levels of morbidity and mortality, but mainly because these are strongly affecting the younger age ranges^(^ 7 ^)^. It is essential that practical methods for identifying this initially-silent syndrome should be outlined. Thus, the researcherss aimed to identify the frequency of the components of metabolic syndrome among university students. 

## Methods

This descriptive study was undertaken with 550 students of a public higher education teaching institution located in the municipality of Picos in the Brazilian state of Piauí (PI), the sample being calculated with the use of the formula for a finite population. 

The inclusion criteria were: to be at least 18 years old, to accept to participate in the study, to be enrolled at, and to regularly attend, the University and to have participated in all the stages of the study (interview and collection of samples).

Data collection took place in January - March 2013, through the administration of a questionnaire soliciting socioeconomic data, lifestyle (smoking, drinking and practicing of physical activity) and components of MetS, such as the measurement of Abdominal Circumference (AC) and Arterial Pressure (AP), as well as the collection of blood samples for the evaluation of serum levels of venous glycemia, triglycerides, and HDL-cholesterol.

For the classification of the MetS components, the researchers used the National Cholesterol Education Program´s Adult Treatment Panel III (NCEP-ATP III)^(^ 2 ^)^, which proposes the aggregation of at least three of the five risk factors, with highly specific cut-off values, such as: abdominal circumference > 88 cm in women and > 102 cm in men, triglycerides ≥ 150 mg/dl, HDL - cholesterol < 40 mg/dl in men or < 50mg/dl in women, arterial pressure ≥ 130/85 mmHg and circulating glucose ≥ 110 mg/dl.

A 12 hour fast was followed, and a laboratory was contracted to this end. The collection of blood samples occurred on the premises of the university itself. 

In relation to lifestyle, those students who did not practice light or moderate activity for a minimum of 30 minutes daily at least five days per week, or 20 minutes a day of vigorous activity on three or more days per week, were classified as sedentary. The following are considered light or moderate activities: walking, walking on a treadmill, lifting weights, hydrogymnastics, gymnastics in general, swimming, martial arts, cycling and volleyball. Vigorous activities were running, running on a treadmill, aerobic gymnastics, football, basketball, and tennis^(^ 8 ^)^.

In relation to smoking, the students were classified in four categories: daily smokers, occasional smokers, ex-smokers, and non-smokers. Those who smoked at least one cigarette per day at least one month prior to filling out the questionnaire were considered to be daily smokers; occasional smokers were those who did not smoke daily; ex-smokers were those who, after having been smokers, had ceased smoking at least one month previously; and those who had never smoked, or who had been smoking for less than one month, were considered non-smokers^(^ 9 ^)^.

In relation to drinking alcohol, the Alcohol Use Disorders Identification Test (AUDIT) was used, a test of 10 questions developed by the WHO as a tracking instrument specifically for identifying people with harmful consumption of alcohol, as well as those who have already developed dependence^(^ 10 ^)^.

Analysis of the data was undertaken using the Statistical Package for the Social Sciences IBM (SPSS), version 20.0; the statistical measurements were calculated (means and standard deviation), as were the Pearson Chi-Square and the Fisher-Freeman-Halton test for association of the variables. For all the inferential statistical analyses, those with p < 0.05 were considered statistically significant. 

The undertaking of the study met the national and international rules for ethics in research involving human beings, and was approved by the research ethics committee of the Federal University of Piauí under Protocols N. 0408.0.045.000-11.

## Results

The sample consisted of 550 university students, of whom 66.2% were female, with a mean age of 22.6±4.41, of whom 85.1% were in the age range between 18 and 25 years old. 51.6% stated that they were of mixed race. 

In relation to economic class, 51.8% were denominated between the classes C1 and C2, with a mean income of R$1,629.00.

Table 1 refers to the distribution of the students in relation to lifestyle. 

**Table 1 -:** Distribution in relation to the lifestyle of the students of a public university. Picos, PI, Brazil, 2013

Variables		n	%
Physical activity			
	Active	156	28.3
	Sedentary	394	71.7
Smoking			
	Has smoked one cigarette per day for at least one month	10	1.8
	Does not smoke on a daily basis	32	5.8
	Stopped at least one month ago	03	0.6
	Has never smoked or started less than one month ago	505	91.8
Drinking alcohol			
	Zone I – low risk	197	35.8
	Zone II – medium risk	267	48.5
	Zone III – high risk	63	11.5
	Zone IV – Alcohol Dependence Syndrome	23	4.2

In relation to lifestyle, 71.7% were sedentary. 91.8% stated that they had never smoked or had been smoking for less than one month, and 48.5% were classified in zone II, of medium risk. 

Table 2 describes the anthropometric data of Abdominal Circumference and the other components of Metabolic Syndrome. 

**Table 2 -:** Anthropometric data and components of metabolic syndrome (NCEP-ATP III, 2001) among students of a public university. Picos, PI, Brazil, 2013

Variables		n	%	Min-Max	Median	Mean±SD*
Abdominal circumference				57-124	77	783±10.3
	Normal	518	94.2			
	Raised	32	5.8			
Fasting blood glucose				67-178	82	83.3±11.9
	Normal	543	98.7			
	High	07	1.3			
Triglycerides					116	122.2±55.0
	Normal	446	81.1	64-567		
	High	104	18.9			
HDL cholesterol^†^				22-104	42.5	43.9±8.0
	Normal	195	35.5			
	Low	355	64.5			
Blood pressure				SAP^‡^: 80-175	110	SAP^‡^: 109.9±12.1
	Good	369	67.1	DAP^§^: 60-110	70	DAP^§^: 69.9±9.7
	Normal	133	24.2			
	Borderline	48	8.7			
BMI^||^				17-47.5	22.2	26.7±94.0
	Normal	438	79.6			
	Raised	112	20.4			

* Standard Deviation

† High Density Lipoprotein

‡ Systolic Arterial Pressure

§ Diastolic Arterial Pressure

|| Body Mass Index.

According to the anthropometric data, 5.8% had raised abdominal circumference, and 20.4% had excess weight (overweight/obesity). In relation to the biochemical data, 1.3% had raised fasting blood glucose levels, 18.9% had a high level of triglycerides, and 64.5% had low HDL cholesterol. 8.7% had blood pressure levels compatible with borderline high blood pressure.

Table 3 describes the anthropometric data and other components of MetS and their relationship to sex.

**Table 3 -:** Anthropometric data and components of metabolic syndrome (NCEP-ATP III, 2001) among students of a public university. Picos, PI, Brazil, 2013

Variables	Total Mean ± SD^†^	Female Mean ± SD^†^	Male Mean ± SD^†^	p*
Abdominal circumference	78.3 ± 10.3	74.6±7.9	85.4±10.9	0.000
BMI^‡^	26.7 ± 4.0	27.9±15.6	24.5±4.6	0.638
Fasting blood glucose	83.3 ± 11.9	83.1±11.2	83.8±13.3	0.184
Triglycerides	122.2 ± 55.0	113.3±42.8	139.9±70.1	0.002
HDL cholesterol^§^	43.9 ± 8.0	44.3±8.6	43.3±6.8	0.862
Systolic arterial pressure	109.9 ± 12.1	105.3±9.4	119.0±11.6	0.000
Diastolic arterial pressure	69.9 ± 9.7	68.4±8.3	72.9±11.6	0.000

* t-student

† Standard deviation

‡ Body Mass Index

§ High Density Lipoprotein

In the test of comparison of the MetS components' means with sex, it is observed that there was a difference between the means related to abdominal circumference, to the triglycerides and to systolic and diastolic arterial pressure (p<0.05).

Table 4 presents the relationship between the socioeconomic variables and lifestyle variables, with the components for metabolic syndrome. 

**Table 4 -:** Relationship of socioeconomic variables and lifestyle variables with components for metabolic syndrome, among students of a public university. Picos, PI, Brazil, 2013

Variables		No risk factor n (%)	1 to 2 risk factors n (%)	3 to 5 risk factors n (%)	p*
Age range					0.000
	18-25	103 (22.0)	356 (76.0)	9 (2.0)	
	26-51	10 (12.2)	62 (75.6)	10 (12.2)	
Sex					0.000
	Female	57 (15.7)	299 (82.1)	8 (2.2)	
	Male	56 (30.1)	119 (64.0)	11 (5.9)	
Color					0.744
	White	39 (21.2)	137 (74.4)	8 (4.3)	
	Black	12 (18.8)	50 (78.1)	2 (3.1)	
	Asian	-	18 (100.0)	-	
	Mixed European and African descent	62 (21.8)	213 (75.0)	9 (3.2)	
Economic class					0.178
	A1 - A2	2 (18.2)	9 (81.8)	-	
	B1 - B2	40 (21.5)	135 (72.6)	11 (5.9)	
	C1 - C2	53 (18.6)	226 (79.3)	6 (2.1)	
	D – E	18 (26.5)	48 (70.6)	2 (2.9)	
Physical activity					0.887
	Active	35 (22.4)	116 (74.3)	5 (3.2)	
	Sedentary	78 (19.8)	302 (76.7)	14 (3.5)	
Drinking alcohol					0.106
	Low/medium	92 (19.8)	358 (77.1)	14 (3.1)	
	High/ADS^†^	21	60	5	
Smoking					0.106
	Smokes/not daily	10 (23.8)	30 (71.4)	2 (4.8)	
	Quit/never smoked	103 (20.3)	388 (76.4)	17 (3.3)	

* Pearson Chi-Square

† Alcohol Dependence Syndrome

In relation to the combination of the socioeconomic variables and lifestyle variables with the number of components of MetS, it is ascertained that there was a statistically significant association with sex and age range (p<0.05).

Hence, taking into consideration the components for MetS indicated in the NCEP-ATP III, 20.5% of the sample did not present any component of MetS, while 64.4% presented at least one, 11.6% had two components, and 3.5% had three or more. 

## Discussion

The investigation in question assessed 550 university students, of both sexes, with a mean age of 22.6±4.41. 71.7% were sedentary, 1.8% confirmed that they smoked, and 48.5% were classified as at medium risk for alcoholism. Furthermore, 5.8% had a high abdominal circumference and 20.4% had excess weight. 1.3% and 18.9% had raised fasting blood glucose and triglycerides, respectively. 64.5% had low HDL cholesterol and 8.7% had blood pressure levels compatible with borderline high blood pressure. Thus, out of the sample, 64.4% had at least one component for MetS, 11.6% had two, and 3.5% had three or more. 

The variety found in the prevalence of MetS results from the criteria found in the literature and in the cutoff points determined by the authors. A separate investigation undertaken with university students in the city of Fortaleza in the state of Ceará (CE) indicated that in spite of the low prevalence of MetS among the university students (1.7%), the majority had at least one or two isolated components. Of the students with three or more components, emphasis was placed on the males, who had higher mean blood pressure and levels of triglycerides. In addition, excess weight (overweight and obesity) was directly associated with a greater presence of components of MetS^(^ 11 ^)^. Similar findings were found in this study, in which the prevalence of three or more components was only 3.5%, and the male sex presented a greater presence of these components (p<0.05).

An investigation undertaken with 41 students at Unibrasil, in order to identify risk factors for the development of Metabolic Syndrome (MetS) and cardiovascular disease found that 24.4% of the students studied had MetS, by the simple fact that they presented Abdominal Circumference (AC) above normal reference values. 7.3% presented two risk factors, 21.9% presented one risk factor, and 46.34% of the individuals did not present risk factors for the development of MetS^(^ 12 ^)^.

Excess weight is emphasized when one investigates MetS. One study with university students from São Paulo identified a prevalence of 28.6% and 55.6% of overweight and 22.4% and 5.5% obesity, respectively, in women and men. It also evidenced that there was significant variation in glycemia, HDL-c, and maximum arterial pressure between the sexes. There is a high prevalence of deviations in the levels of Low Density Lipoproteins (LDL-c) (81.6% of the women and 88.9% of the men), in the reduced levels of HDL-c (49% of the women and 83.3% of men) and high prevalence of women with rises in AP (75.9%)^(^ 13 ^)^. In the present study, on the other hand, the highest alterations were perceived in the male sex, in relation to AC, triglycerides and AP.

One study aiming to investigate the prevalence of metabolic syndrome in young adults and the influence of birth conditions and nutritional status in adolescence found a prevalence of MetS of 13% of those assessed. The birth conditions did not present a relationship with the determination of the syndrome. Those diagnosed with MetS presented - in adolescence - higher values for weight (11 kg; p =<0.001), waist circumference (8 cm; p < 0.001) and body mass index (2.5 kg/m^2^; p= 0.002)^(^ 14 ^)^.

The use of other methods of selecting cohorts can lead to changes in the prevalence of MetS. One study with patients ≥18 years old, in a specialized nutrition unit in the University Hospital of the Federal University of the State of Rio de Janeiro (UNIRIO) estimated the prevalence of MetS by the NCEP-ATP III and International Diabetes Federation (IDF) criteria among 414 patients, the comparison being made by the agreement percentage and kappa. The prevalence by the IDF criteria was slightly superior to that of the NCEP (61.1% vs 55.6%), with agreement of 93%, kappa=0.855 (p value X^2^=0.000). It was observed that MetS increases with age, without a significant difference between the sexes, as well as with the rise in the body mass index^(^ 15 ^)^.

Studies with other populations, such as children and adolescents, have also been mentioned comparatively, due to the few studies evidenced with university students. Emphasis is placed on one survey undertaken in the city of Viçosa, in the state of Minas Gerais, with 99 adolescents aged between 10 and 19 years old, attended by the Adolescent Health Care Program, which pointed to the prevalence of the syndrome in 16.6% of the sample, although when only the adolescents with excess weight were considered, the prevalence was 35.5%,^(^ 16 ^)^ a data far higher than those found in this and in other studies with university students^(^ 11 ^,^ 13 ^)^.

One study aiming to describe the prevalence of metabolic syndrome in an outpatient sample of 74 children and adolescents with overweight and obesity identified that the anthropometry revealed a population with the presence of obesity (70.3%), of whom 34 (45.9%) were seriously obese and 22 (29.7%) were morbidly obese. In relation to the serum levels of TG, HDL-cholesterol and glycemia, the most prevalent metabolic alteration was hypertriglyceridemia, varying from 66.2 - 74.3%. Among the other components, on the other hand, the waist circumference (WC) was high in 17 (100%), while fasting blood glucose presented a lower prevalence of alteration (1.4 - 2.7%)^(^ 17 ^)^. Levels of excess weight (overweight/obesity) were found in a lower proportion in this study, and the fasting blood glucose also presented lower alterations.

One study undertaken with 390 male and female adolescents aged between 10 and 14 years old, with the aim of analyzing the relationship between obesity and metabolic syndrome, found that in the nutritional condition, the male sex presented obesity of 27.1%, overweight of 4.3%, and 68.6% normal weight. Regarding the female sex, 29.5% have normal weight, 1.6% are overweight, and 68.9% are obese. Obesity in the female sex was statistically greater than among the males. The prevalence of MetS was 37%, and the males had a greater prevalence of metabolic syndrome. No statistical difference was found between the sexes in the following variables: triglycerides, HDL-c and arterial pressure^(^ 18 ^)^. In the present investigation, in relation to sex, there was a difference between the means related to abdominal circumference, to the triglycerides, and to systolic and diastolic pressure (p<0.05).

One descriptive study undertaken with females aged between 12 and 18 years old, from a college in Ribeirão Preto, was divided into groups: overweight/obesity (n=30) and control (normal weight) (n=39) and indicated that the adolescents with overweight/obesity presented greater levels of arterial pressure, glucose, triglycerides, uric acid, PAI-1, fibrinogen and insulin, and lower levels of HDL cholesterol, in relation to the control group. The analysis of risk factors demonstrated that 76.7% of the adolescents from the overweight group presented two or more risk factors related to metabolic syndrome, while 79.5% of the adolescents from the control group presented no alterations or only one^(^ 19 ^)^. In the present study, 71.7% of the sample were sedentary, 5.8% had raised AC, 1.3% had raised glycemia, 18.9% had a raised level of triglycerides, 64.5% had low HDL cholesterol, 8.7% had borderline high blood pressure and 20.4% had BMI classified as overweight/obesity.

In relation to the biochemical alterations, 73.4%; 44.7%; 49.7%; 41.2% and 5.5% presented alterations in the levels of total cholesterol, LDL, High Density Lipoproteins (HDL), triglycerides and fasting blood glucose, respectively^(^ 16 ^)^. In this study, 5.8%, 1.3%, 18.9%, 8.7%, 20.4%, and 64.5% presented higher levels of AC, venous glycemia, triglycerides, AP, BMI and low level of HDL, respectively. 

In an investigation undertaken with university students in the city of Fortaleza (CE), on the other hand, it was evidenced that high values of triglycerides, total cholesterol and cholesterol associated with low-density lipoprotein were found in 23.0%, 9.7%, and 5.9% of the students, respectively^(^ 20 ^)^.

The results found in this work have important implications for public health, as these risk factors in young adults (the majority of the sample) are associated with the possible presence of metabolic syndrome in the future. In the light of this, the need is emphasized to deepen the studies for establishing a diagnostic criteria, making it possible to undertake strategies aiming to control and prevent metabolic disorders, such that these strategies may have a positive impact on the cardiovascular diseases in the future. 

This study's limitations, which include the sampling process adopted, and the sample fraction recruited, with a possible absence of population representativity of the universe of university students, require prudence in interpreting the results obtained. Furthermore, studies with university students are operationally more complex, principally regarding the collection of blood samples, as many had placements or worked until late at night, and had to eat - and thus did not comply with the 12 hours of fasting. On the other hand, this investigation could contribute to emphasizing the methodological difficulties in determining alterations which depict the situation of MetS among university students. 

## Conclusion

Although the data analyzed belonged to a specific group - in this case, university students - the results demonstrate the high prevalence of excess weight, of sedentarism, and of alterations in these individuals' lipid profile, as the majority of the study subjects presented from 1 to 2 components for MetS. These factors may represent a risk for metabolic syndrome and future cardiovascular diseases. 

This profile reinforces the importance of the early diagnosis of, and the monitoring of, these alterations in the target population, with the aim of reducing the risk of developing chronic comorbidities; at the same time, they may serve as support for clinical practice and for planning public health policy actions. 
